# Tuberculous Subclavian Artery Pseudoaneurysm in a Young Male With Hemoptysis

**DOI:** 10.7759/cureus.75638

**Published:** 2024-12-13

**Authors:** Ana Raquel Soares, Sofia Eusébio, Pedro Fiúza, Tiago Pack, Tiago F Ribeiro

**Affiliations:** 1 Internal Medicine, Unidade Local de Saúde São José, Lisbon, PRT; 2 Vascular Surgery, Unidade Local de Saúde São José, Lisbon, PRT

**Keywords:** aneurysm, hemoptysis, mycotic pseudoaneurysm, subclavian artery, tuberculosis

## Abstract

Subclavian artery pseudoaneurysms (SAPs) are rare and most often secondary to trauma. On the contrary, a mycotic origin is exceedingly rare, and defining this etiology can become challenging. We present a rare case of a tuberculous SAP in a young patient.

A 19-year-old male patient with no past medical history and no relevant epidemiological context presented to the ED with three-day left pleuritic thoracalgia and hemoptysis. A chest roentgenogram revealed a left paratracheal opacity, and a CT angiography revealed a voluminous left SAP. Accordingly, endovascular surgical treatment of the SAP was performed, with complete symptom remission. Although mycotic etiology was suspected, the first microbiological assays were negative. Following six months asymptomatic, hemoptysis recurred, and a bronchoscopy was performed. Mycobacterial cultures of bronchoalveolar lavage were positive for *Mycobacterium tuberculosis*, and tuberculous left SAP and pulmonary tuberculosis were ultimately confirmed. Treatment with first-line anti-tuberculosis drugs was completed without associated complications and with symptom resolution.

This case highlights that in the presence of a non-traumatic arterial pseudoaneurysm, the diagnosis of tuberculosis should always be considered and carefully investigated. An approach with a combination of anti-tuberculous therapy and surgery seems the most appropriate in these cases. When endovascular treatment is performed, follow-up must be maintained to exclude future complications, particularly those related to possible infection of the prosthetic vascular material.

## Introduction

Subclavian artery aneurysms (SAAs) are rare, corresponding to about 1% of peripheral arterial aneurysms [1,2], although the exact incidence in the general population is unknown. From a historical perspective, Coselli and Crawford reported and treated 18 aneurysms of the intrathoracic segment of the subclavian artery between 1969 and 1986 [3].

Regarding etiologies, SAA may occur due to atherosclerosis, thoracic outlet syndrome, collagen disorders, infection, trauma, or iatrogenic injuries [1,4]. Atherosclerosis and thoracic outlet syndrome are the main causes of true SAA, and pseudoaneurysms are mostly secondary to trauma, often iatrogenic [1]. Mycotic SAAs are related to an infection of the vessel wall and are very rare [1]. Defining the etiology of the aneurysm can be difficult but is always important, as it can have implications in terms of treatment and prognosis.

We present a rare case of a tuberculous subclavian artery pseudoaneurysm (SAP) in a young patient with no relevant past medical history or epidemiological context who successfully underwent endovascular repair and anti-tuberculosis medication.

## Case presentation

**Figure 1:**
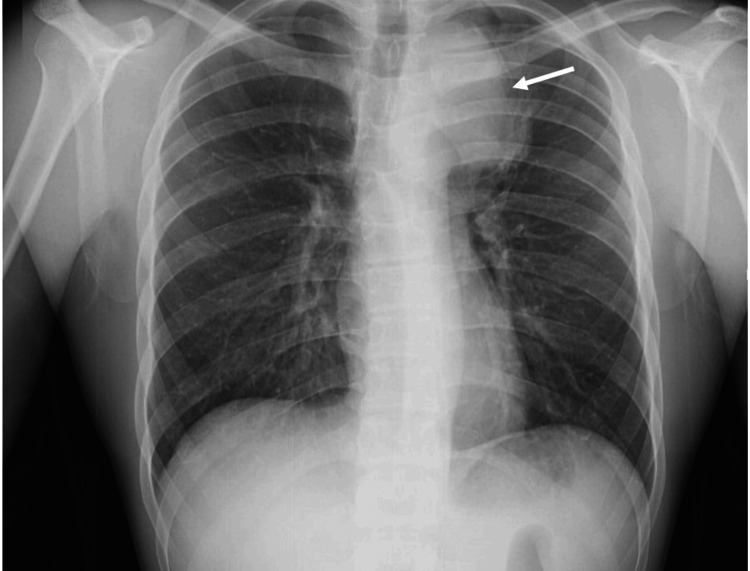
Left paratracheal opacity on chest roentgenogram (white arrow)

A 19-year-old male patient with no past medical history presented to the ED with three-day left pleuritic thoracalgia and hemoptysis. On physical examination, he presented with sinus tachycardia (120 beats per minute) and was otherwise unremarkable. Blood tests at admission unveiled D-dimer elevation (711 µg/L) and C-reactive protein elevation (42 mg/L); no other changes were observed. A chest roentgenogram revealed a left paratracheal opacity (Figure 1).

**Figure 2:**
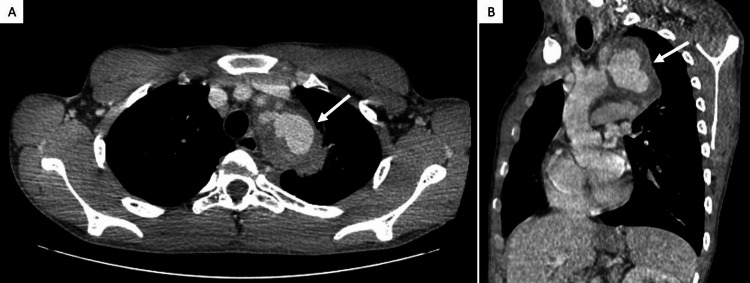
CT angiography revealing left subclavian artery pseudoaneurysm (white arrows) (A) Axial view and (B) sagittal view

CT angiography was performed, and a voluminous left SAP with 70 x 52 mm (Figure 2) and perivascular stranding was noted. According to these findings, vascular surgery consultation was obtained, and expedited surgical treatment of the SAP was performed by implanting polytetrafluoroethylene stent-graft, with complete symptom remission. After stent-graft implantation, single antiplatelet therapy with acetylsalicylic acid (100 mg per day) was maintained indefinitely.

Further study was carried out, and laboratory investigations, including CBC and autoimmune assays, were within the normal range and negative, respectively. Although the suspicion of a mycotic aneurysm was raised, there was no relevant epidemiological context, and all microbiological assays performed were negative, including virus polymerase chain reaction tests; serology tests for syphilis, Brucella spp, *Borrelia burgdorferi*, *Coxiella burnetti*, *Rickettsia conorii*, and *Mycoplasma pneumoniae*; and hemocultures for Mycobacterium spp.

**Figure 3:**
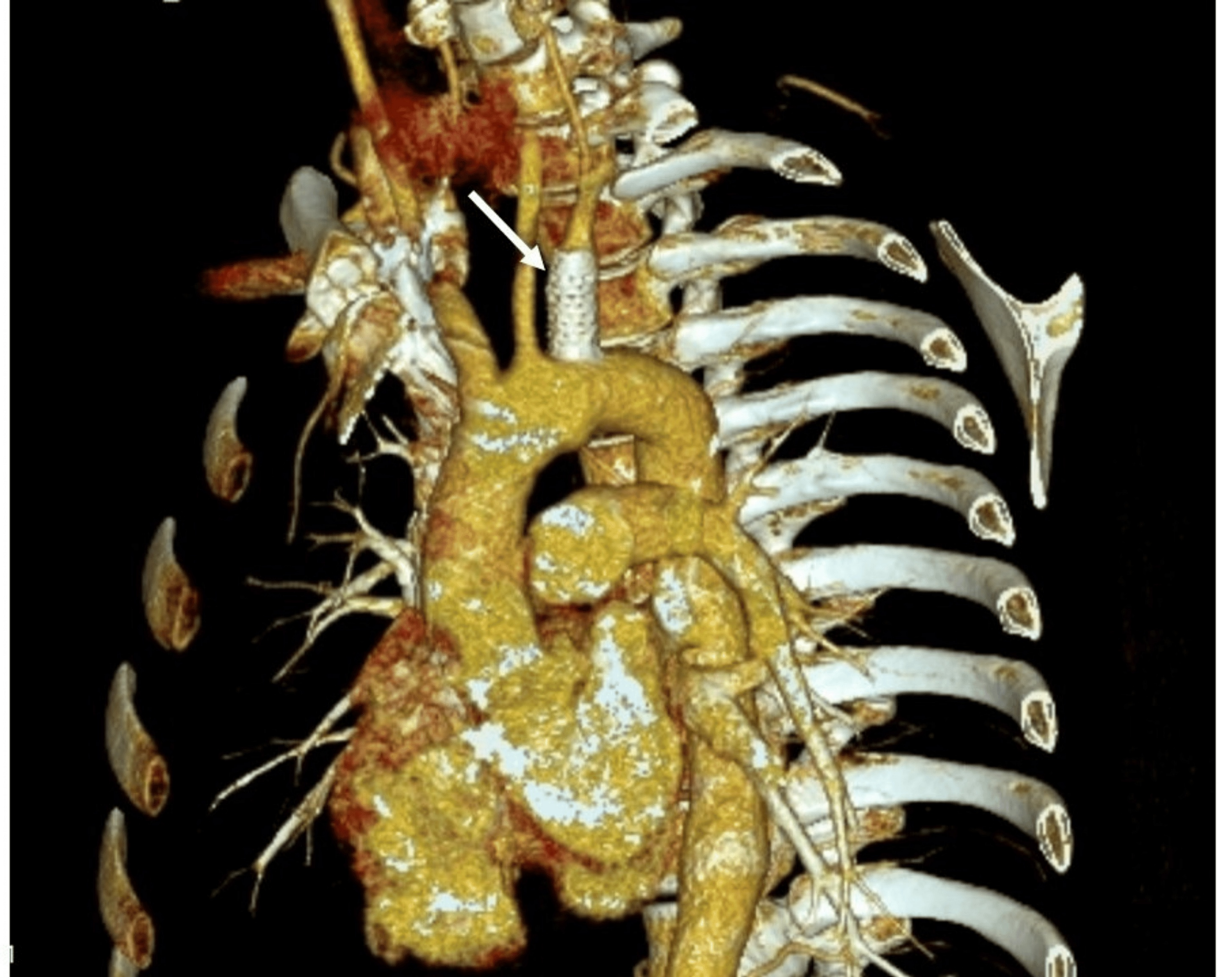
CT angiography depicting patent stent-graft covering subclavian pseudoaneurysm (white arrow) Three-dimensional volume-rendering reconstruction

**Figure 4:**
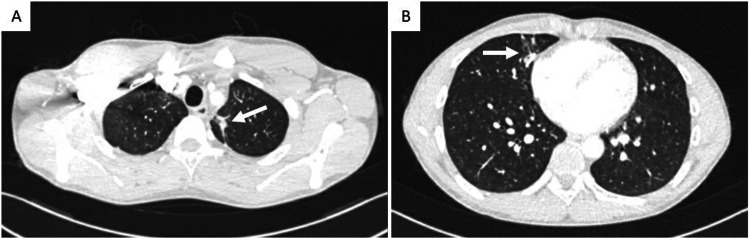
CT revealing residual lung densifications in the left upper lobe and the inner segment of the middle lobe (white arrows) Axial view

The patient remained asymptomatic for six months when hemoptysis recurred. On CT angiography, no local vascular complications were detected (Figure 3), while residual lung densifications were evident in the left upper lobe and the inner segment of the middle lobe (Figure 4). Bronchoscopy was deemed necessary to exclude lesions of the trachea or bronchi that could cause hemoptysis and collect samples for microbiology, considering again the hypothesis of mycotic SAP.

Following negative acid-fast bacilli smear and molecular nucleic acid amplification tests on bronchoalveolar lavage obtained from the middle lobe, mycobacterial cultures were positive for *Mycobacterium tuberculosis*. Tuberculous left SAP and pulmonary tuberculosis were then confirmed.

Resistance to first-line anti-tuberculosis drugs has not been documented, so the patient completed treatment for two months with isoniazid (300 mg per day), rifampicin (600 mg per day), pyrazinamide (1500 mg per day), and ethambutol (1200 mg per day), followed by seven months of isoniazid (300 mg per day) and rifampicin (600 mg per day), according to national recommendations, without associated complications and with symptoms resolution. After almost four years of follow-up, the patient remains asymptomatic, with no signs of active infection and no vascular abnormalities.

## Discussion

Tuberculous arterial aneurysms are exceedingly rare and are associated with a significant risk of rupture [5,6]. In this setting, *Mycobacterium tuberculosis* bacilli may colonize the arterial wall, subsequently eroding its thickness, leading to caseating necrosis of the arterial wall. Therefore, these present frequently as pseudoaneurysms [6,7]. Most often, these affect the thoracic and abdominal aorta, with a minority affecting limb arteries. Contiguity with the site of tuberculosis infection is the most common mechanism underlying these aneurysms, as well as a predictor of aneurysm rupture, as was found in this patient [5].

The rupture rate of tuberculous aneurysms is higher than other mycotic aneurysms, and these ruptures often lead to fatal bleeding [5]. This highlights the importance of early diagnosis and timely treatment, aiming to prevent this potentially life-threatening complication. Notwithstanding, early identification of tuberculosis may be difficult in such cases. While associated with pulmonary tuberculosis, interestingly, about 40% of these have no ongoing clinically evident infection [5]. When symptomatic, most present chest and/or shoulder pain or a pulsatile mass in the supraclavicular region [1], which might be accompanied by mass effect or hemorrhage. The last, in the form of hemoptysis, was the predominant clinical feature in this case. In patients initially diagnosed with active tuberculosis, in whom mass lesions appear or if clinical deterioration occurs, vascular lesions such as aneurysms must be excluded when there is a high index of suspicion.

There are a few described cases of hemoptysis caused by subclavian pseudoaneurysms [4,8]. In this case, both the pulmonary lesions caused by tuberculosis and the pseudoaneurysm, which is large and causes associated lung damage, may have contributed to hemoptysis.

Tuberculous aneurysms should be treated with combined therapy, including surgery and anti-tuberculosis medications, even if the aneurysms are small [5]. Endovascular treatment of SAP, as performed in this case, has been considered safe and effective [9], although that is not so clear regarding infectious aneurysms. The decision regarding the type of treatment must always be individualized.

Following infective aneurysm repair with prosthetic material, there is an inherent lifelong risk of vascular graft infection. Notwithstanding, in the absence of clinical, laboratory, or radiographic findings suggesting otherwise, there is no evidence supporting different anti-tuberculosis regimens.

## Conclusions

Tuberculous arterial aneurysms are rare, and the diagnosis of tuberculosis can be challenging, particularly in the absence of constitutional symptoms. Notwithstanding, considering the increase in the global incidence rate of tuberculosis in recent years, the incidence of tuberculous aneurysms may also increase. When there is evidence of a non-traumatic arterial pseudoaneurysm, the diagnosis of tuberculosis should always be considered and investigated to allow timely and appropriate treatment in order to prevent future complications.

Despite little evidence regarding the treatment of this pathology, an approach with a combination of anti-tuberculous therapy and surgery seems the most appropriate. When endovascular treatment is performed, follow-up must be maintained to exclude future complications, particularly those related to possible infection of the prosthetic vascular material.
